# A methodological perspective on learning in the developing brain

**DOI:** 10.1038/s41539-022-00127-w

**Published:** 2022-06-02

**Authors:** Anna C. K. van Duijvenvoorde, Lucy B. Whitmore, Bianca Westhoff, Kathryn L. Mills

**Affiliations:** 1 Institute of Psychology, Leiden University, Leiden, The Netherlands; 2 Leiden Institute for Brain & Cognition, Leiden University, Leiden, The Netherlands; 3 Department of Psychology, University of Oregon, Eugene, OR USA; 4 PROMENTA Research Center, Department of Psychology, University of Oslo, Oslo, Norway

**Keywords:** Cognitive neuroscience, Human behaviour

## Abstract

The brain undergoes profound development across childhood and adolescence, including continuous changes in brain morphology, connectivity, and functioning that are, in part, dependent on one’s experiences. These neurobiological changes are accompanied by significant changes in children’s and adolescents’ cognitive learning. By drawing from studies in the domains of reading, reinforcement learning, and learning difficulties, we present a brief overview of methodological approaches and research designs that bridge brain- and behavioral research on learning. We argue that ultimately these methods and designs may help to unravel questions such as why learning interventions work, what learning computations change across development, and how learning difficulties are distinct between individuals.

## Introduction

Childhood and adolescence are considered natural times of learning and adjustment. In the first decades of life, changes in physical appearance, as well as cognitive and social-emotional development, are readily apparent^1–4^. What is, however, less readily observed are the profound changes in the structure, function, and connectivity of the brain. These changes underlie the development of skills and cognitive processes, for which input from the environment (i.e., experience) is required to fully develop.

Learning, defined as the gain of knowledge or skills through experience, can take on many different forms and can be studied at multiple levels. For instance, cognitive domains of learning—such as learning to read, or the ability to learn through feedback—can be studied together with measures of brain development to eventually inform our understanding of how children and adolescents learn in environments such as school and society. In this review, we discuss three methodological approaches with clear potential for advancing the study of learning in relation to the developing brain.

We will start this review with a concise overview of how the brain is changing over time in structure and function, and the role of plasticity in learning. We will first highlight the opportunities afforded by longitudinal intervention designs, which can inform approaches to understand learning processes during periods of substantial brain development. We then discuss a computational modeling approach that examines how people adjust to their environment based on the processing of positive and negative outcomes. In computational models, learning processes can be broken down into several steps—cognitive computations—that may help to formalize the process of learning and can be linked to underlying brain development. Finally, we highlight the importance of recognizing that developmental patterns differ across individuals when examining learning. As development is not the same for everyone, subgroups of individuals may share learning strategies or even struggles in learning. We will discuss methodological approaches that allow us to study individual differences in learning, and the degree to which these differences in learning are reflected in the developing brain.

Taken together, we present a developmental cognitive neuroscience perspective on child and adolescent learning by summarizing different methodological approaches and their potential combination with brain imaging techniques, such as magnetic resonance imaging (MRI).

### Structural and functional brain development

Methods such as MRI have greatly advanced the study of the development of the human brain. The first years of life are characterized by a vast increase in cortical grey matter^5^, which is composed of neural and glial cell bodies, dendritic processes and synapses, as well as blood vessels. This initial overgrowth is followed by a reduction in cortical grey matter volume between roughly ages 8–25 years^6,7^. While the resolution of MRI does not allow for us to identify specific cellular changes occurring, the observed reduction in cortical grey matter during childhood and adolescence is thought to, in part, reflect *synaptic pruning*, in which the brain is cutting back and reorganizing synaptic connections potentially on the basis of how frequently they are used^6,7^. This reduction in cortical grey matter is a normative developmental pattern that can vary across individuals and also varies across regions of the cortex^8,9^. Another structural brain measure, white matter volume, is composed of the myelinated axons connecting distal regions of the brain and increases until mid-adolescence or early adulthood before it begins to stabilize^6^. This increase in white matter volume reflects, in part, the strengthening of synaptic connections between brain regions^1,10^. In short, these changes in the brain result in a more efficient and specialized brain system, with stronger connections between brain regions.

These developmental changes in brain *structure* are paralleled by changes in brain function and behavior in the affective, cognitive, and social domains^1,11,12^. Brain *function* is typically studied in task-based paradigms, examining individuals’ brain responses to a specific event, such as receiving negative feedback or a rewarding outcome. In addition to task-based paradigms, the intrinsic functional connectivity of the brain has been studied during scans called ‘resting-state’. In these scans, participants’ spontaneous brain activation is examined while they are lying in the scanner (but without falling asleep). Resting-state fMRI analyses are designed to probe functional brain *connectivity*. Functional brain connectivity refers to the correlation of activation signals between different brain regions or networks and is thought to signal to what extent these regions/networks are functionally related. Developmental studies have observed changes in functional connectivity strength, and changes in network interactions^13^. For instance, functional connectivity strength between subcortical and cortical brain regions increased with age^12^, and are tied to changes in reward-sensitivity and learning. Resting-state studies have shown that the brain becomes increasingly ‘modular’ (i.e., functionally segregated) across childhood and adolescence^14,15^. An increase in modularity is also seen in structural brain development^16^, and both these functional and structural network-level changes are shown to support the development of, for instance, executive functioning^14,16^. Resting-state and task-based studies can thus both provide unique insights into the development of a specific cognitive function or experimental manipulation, as well as the broad network-organization of the brain.

Although not the main focus of this article, when discussing the developing brain and learning, the importance of plasticity (i.e., the brain’s *ability* to change and adapt as a result of experience) is evident^17^. A typical distinction is made between brain plasticity that is *experience-independent*, in which brain changes unfolds, relatively independent of experience; brain plasticity that is *experience-expectant*, in which neural sensitivity is attuned to particular environmental stimuli during specific developmental windows (i.e., sensitive periods); and brain plasticity that is *experience-dependent*, which reflects experiences and environmental inputs that can vary between individuals and supports learning throughout life^18^. Neuroplasticity, particularly in sensitive periods, is related to molecular processes that can inhibit or stimulate brain plasticity through neurotrophic factors such as brain-derived neurotrophic factor (BDNF) that may result in changes in synaptic and neural pruning^19^.

An example of experience-expectant learning in the brain comes from the development of basic sensory processing regions. Seminal research on visual processing has demonstrated that the development of the visual cortex is dependent on relevant stimulation from the environment. Specifically, depriving visual input to one eye resulted in ocular dominance in the visual cortex of the eye that received input, and an absence of developing binocular vision^20^. Moreover, this work highlighted that the impact of experience on visual cortex development depended on the environmental input in a specific developmental window, referred to as a *sensitive period*. Sensitive periods are periods of heightened neuroplasticity to specific environmental input. They have been historically studied for the development of the visual neural system, yet are thought to occur across multiple cognitive and social-emotional domains such as language and face processing^17,21^. Experience-dependent learning, on the other hand, can be thought of as learning due to practice, exposure, or experience. This type of learning therefore depends on individual’s experiences and can occur at all points of ontogeny. Experience-dependent learning may contribute to brain development by gradually modulating brain connectivity, activation, or structure. The developmental cognitive neuroscience work we will discuss here focuses on experience-dependent learning in the brain across childhood and adolescence.

### Learning shapes the developing brain: language

Although brain development may affect the efficiency with which we can learn about the world, or acquire a new skill, learning can also have an impact on brain structure and function at a level that can be measured through MRI, through observed changes in cortical thickness and changes in functional activation during task or at rest. Given that learning in cognitive domains often occurs at the same time as substantial development in both brain structure and function, it is a challenge to differentiate whether the observed changes in the brain reflect *experience-independent* maturational processes, or *experience-dependent* learning. In order to specify the areas of the brain that support learning in cognitive domains, we must be able to disentangle if an observed effect is related to the experience and not just reflective of maturational change that might occur in absence of the experience of learning a specific skill.

By taking an example of one skill that has to be explicitly taught in order to be learned, reading, we can begin to unpack these processes. The left arcuate fasciculus and inferior longitudinal fasciculus are white matter tracts that are considered crucial for skilled reading^22,23^. The left arcuate fasciculus connects anterior and posterior brain regions (i.e., frontal, parietal, and temporal lobes). The inferior longitudinal fasciculus connects the occipital lobe, important for vision, with the temporal lobe, which is—among others—important for semantics. In general, these two white matter tracts continue to mature during the same time as children develop reading skills across childhood and into adolescence. Longitudinal MRI studies have demonstrated substantial increases in fractional anisotropy (FA) in these two tracts from early childhood and into early adolescence^24,25^. This increase in FA is thought to reflect increased integrity of the white matter fibers. This may increase the potential for communication, and thus signaling between the brain regions connected by these tracts. Increases in FA within these tracts have been associated with improvements in reading skill in children^26^, and the rate to which the arcuate fasciculus and inferior longitudinal fasciculus increases in FA across childhood varies by the reading skill of the child^23^. However, in a given MRI study, how can we tell if it is the experience-independent maturational process or the experience of learning that underlies a change in brain measurement between two time points?

Longitudinal intervention designs are one possible method to disentangle maturational and experience-dependent processes in the brain. Although these designs might incur higher costs and burden on participants, repeatedly measuring the same individual over time brings unique opportunities to study ‘true’ development and the influence of experience. Further, the concurrent acquisition of behavioral and neuroimaging measures in these designs allows us to disentangle if change in a brain measure is coupled or uncoupled with change in the behavioral measure of interest. A recent study examined white matter integrity four times in children across the course of an intensive 8-week reading intervention and compared these children to a group of children who did not complete the reading intervention^27^. Participants of the intervention group were recruited based on parent reports of reading difficulties and/or a clinical diagnosis of dyslexia, and the control group was matched for age but not reading level^27^. By taking this longitudinal intervention approach, this study could compare the magnitude of *experience-dependent learning* (in this case, through a reading intervention) on white matter integrity to the magnitude of developmental change. This study identified the left arcuate fasciculus and inferior longitudinal fasciculus as being responsive to the experience-dependent learning in the cognitive domain of reading, as the observed change in integrity of these white matter paths was coupled to improved reading skill level in the children who received the intervention^27^. In contrast, this study also identified white matter paths that predicted a child’s reading skill level, but did not change in white matter integrity throughout the intervention, such as posterior callosal white-matter connections^27^. This finding suggests that some parts of the brain are already related to readiness to learn certain skills even during a developmental period marked by changes in brain structure.

When looking at evidence of learning on brain measures from another cognitive learning domain—second language acquisition—the age period when one learns a second language can result in differential effects on observed brain measures. For example, cortical thickness measures did not differ between monolinguals and bilingual individuals who acquired two languages simultaneously in early life^28^. However, bilingual individuals who acquired their second language later in childhood show differences in cortical thickness of the inferior frontal gyrus (thicker cortex in the left IFG, thinner cortex in the right IFG), and the magnitude of observed differences correlated with the age of second language acquisition^28^. Thus, multilingual individuals showed no difference in overall cortical thickness as long as they acquired their languages simultaneously, and only individuals who acquired another language *later* in life showed a difference in cortical thickness. Given that cortical thickness seems sensitive to experiences, such as later language learning, we must consider what differences in overall measures of cortical thickness could actually represent. Perhaps these group-level differences in cortical thickness changes are more likely to reflect the experience-dependent process of learning, which would be more in line with later second language acquisition, than learning that is expected to occur largely early in life. However, to answer this question, one would need longitudinal designs to compare the magnitude of change in cortical thickness observed in individuals who acquired a second language to magnitude of change observed in individuals of the same age who did not.

### Using a computational modeling approach in the study of learning

One way in which we learn is by processing and integrating the good and bad outcomes we experience. For instance, through positive and negative feedback, we may learn to play a new videogame, learn to play the guitar, or learn the correct spelling of a difficult word. A computational approach can help us to understand these behavioral changes and formalize the process of outcome-based learning in the developing brain.

Computational learning models have been used to investigate questions such as how children, adolescents, and adults learn from positive and negative outcomes and integrate information into subsequent decision-making. An important element of learning in computational reinforcement learning models focuses on the difference between an expected outcome and a received outcome^29,30^. This difference, a so-called *prediction error*, forms the basis of a specific learning signal that indicates how much one should update expectations of the world, and thereby one’s subsequent actions. These prediction error computations have been linked to brain activation, as this learning signal was found to correlate with dopamine release^31,32^ that would instigate neural activation. In the context of understanding learning, reinforcement learning models thus provide a computational link (e.g., a prediction-error signal) between brain-level processes and the observed behavior.

Research examining reinforcement learning in children, adolescents, and adults in combination with the developing brain, has shown that learning signals such as prediction errors are found in brain regions, including the striatum, medial prefrontal cortex, and hippocampus. These brain regions are linked to the processing of reward, value, and memory^33^. A number of studies have investigated differences in prediction-error learning in children, adolescents, and adults to understand sensitivities in learning across development. Some findings highlight that adolescents are particularly sensitive to positive prediction errors (feedback that is better than expected), resulting in higher neural activation in adolescents compared to children and adults in the striatum^34^. Moreover, adolescents outperformed adults in their learning performance, and the strength of functional connectivity between the striatum and hippocampus after positive (compared to negative) outcomes related to subsequent memory performance for positive events. These findings indicate that the striatum, and its closely connected regions (see also^35^), may contribute to heightened reward-learning in some ages, and a bias towards learning from positive outcomes^36^. Note that other studies suggest that the valence-dependency in children’s and adolescent’s learning is context-specific. For instance, it has been observed that adolescents may be more prone to learn from unexpected negative outcomes than adults in other contexts, such as when reward-structures change quickly^37,38^.

Computational models may have several advantages for the study of learning^4,39,40^. A general advantage is that computational models allow to simulate behavior. Generating behavioral data with specific learning parameter settings, allows for better predictions of (expected) behavioral patterns and helps hypothesis testing as well as theory formation. A specific advantage of computational models for brain-behavioral studies is that they allow us to compute latent variables, i.e., variables (such as prediction errors) that cannot be directly observed in behavioral data, but that theory assumes is happening in the brain. These latent variables can be directly linked to brain activation and compared across groups or ages.

Taken together, combining a computational approach with measures of brain functioning allows us to examine learning at different levels of explanation. For instance, on a latent variable level, we can study whether different age groups weigh positive and negative outcomes differently or use different goal-directed strategies in learning^41–43^. On the other hand, developmental change may also occur at the brain (i.e., implementation) level. For instance, learning from experience can involve different brain regions or networks at different ages. Including these levels of explanation in the study of learning can help us to identify mechanisms of learning that may not be apparent, or cannot be disentangled, from observed behavior only.

### Examining heterogeneity in the neurocognition of learning

Another challenge in learning research is to characterize individual differences in learning. Most of the studies on learning, or domains of learning, have focused on comparing brain and behavioral differences between ages or condition. This approach is useful for detecting mean-level differences. However, there may be striking heterogeneity in brain development and learning within groups. How could we target those in the study of learning?

An approach that behavioral studies have taken is to use clustering techniques that detect subgroups in the data. For instance, a recent study used such a data-driven approach and has grouped children based on behavioral measures across a range of learning domains, including reading, phonological processing, and executive functioning^44^. This study showed that within a group of 442 struggling learners, three distinct subgroups were found using this range of behavioral indicators. The first group showed symptoms of elevated inattention and hyperactivity/impulsivity. The second group was characterized by learning problems, and the third by aggressive behavior and disturbed peer relations. Moreover, these groups were distinguished by their structural connectivity of the lateral prefrontal cortex, cingulate cortex, and the striatum. In particular, aggression and peer problems loaded on the integration between the prefrontal cortex and the striatum. These findings support the idea that data-driven profiling can distinguish common learning problems in children and provide insight into the neurobiological mechanisms underlying these problems.

Another study from the same group used a clustering technique on white-matter microstructure in a sample of 313 children and adolescents^45^. This analysis showed that the group with higher white matter integrity in the cingulum had profoundly different cognitive abilities. Applying the cingulate-based grouping to independent groups of typically-developing children and struggling learners showed that children with lower cingulum FA showed lower performance across a variety of cognitive performance measures (e.g., fluid intelligence, working memory, and vocabulary)^45^. The value in this approach may particularly relate to children and adolescents with learning difficulties or psychopathologies that show complex behavioral phenotypes that may be better qualified with brain-based than behavioral subtyping.

A recent study compared sub-grouping profiles generated on behavioral measures (e.g., literacy, numeracy, working memory) and structural brain measures (i.e., regional cortical thickness, gyrification, and sulci depth)^46^. This approach was used on a sample of 479 children and adolescents consisting partly of struggling learners. The results based on behavioral measures indicated six cognitive profiles ranging from high- to poor performers on executive function tasks. A similar profile mapping based on structural brain measures indicated that neural and cognitive mappings for individuals were not one-to-one. That is, the same neural profile could be associated with different cognitive impairments in different children^46^. In a subsequent analysis, the authors observed that an individual’s whole-brain network (i.e., the connectome) of white-matter tracts was more strongly related to the cognitive profiles of struggling learners. Particularly, the hub-like structure of individuals’ brain network related to children’s cognitive abilities. Hubs are well-connected nodes in a network and are therefore assumed play an important role in the communication across a network.

Together, these results aid our understanding how the relationship between brain and cognition may be moderated by the organizational properties of developing brain networks. Consequently, it challenges the idea that a neurodevelopmental disorder (such as learning difficulty) is only linked to one specific neuro-anatomical substrate, and instead suggests that learning difficulties are likely to depend on the interactions and organizational properties between different brain systems^47^. These findings also add to the discussion on the transdiagnostic nature of cognitive developmental problems, in which developmental difficulties in learning reflect complex patterns of associations that are not easily matched to singular diagnostic categories.

Note that these reviewed studies identified subgroups using brain and behavioral measures. Although this approach allows to quantify heterogeneity in learning, brain-behavioral relationships can also be studied at the *individual* level. For instance, finding robust individual-differences markers may help to identify children at risk for developing learning problems. Moreover, questions of heterogeneity in samples have often been tested on cross-sectional datasets with wide age-ranges thereby missing a longitudinal developmental perspective. Finally, these reviewed studies focus on brain structure and not on brain function. Functional brain measures may provide a new mapping for learning profiles, which remains to be explored^47^.

### Where do we go from here?

By drawing from research methods and designs in the domains of reading, reinforcement learning, and learning difficulties, we have presented a brief overview of methodological approaches and key findings in developmental cognitive neuroscience research on learning. We started with the central question of how maturational processes can be distinguished from experience-dependent learning. Longitudinal intervention designs are one possible method to examine learning potential and to disentangle maturational and learning-related processes in the brain. Then, we discussed the use of computational modeling for understanding and disentangling the processes that underlie age-related changes in learning from positive and negative outcomes. Computational modeling approaches are rising in developmental studies, and such studies can move this field forward by quantifying the changes in learning processes over age, and their relation to changes in the developing brain. Finally, we discussed handling individual differences using clustering techniques to find data-driven subgroups that may share a commonality in behavioral learning difficulty, or neural patterns of connectivity and/or brain structure. These findings highlight that not all learning brains are the same, and that methods for detecting individual differences are applicable using brain- and behavioral measurements.

Developmental cognitive neuroscience studies have the potential to advance our understanding of learning by combining innovative research methods with longitudinal datasets capturing development from micro (genes, brain) to macro (behavior, environment) levels^48^. That is, (more) rich longitudinal studies are needed to understand learning and learning challenges within individuals, and to address outstanding questions on how interactions between individual characteristics, experience, and environmental influences shape learning across development^26,48–51^. In the methods discussed in this review, the longitudinal element is sometimes central (such as in intervention studies), whereas in other methodological approaches they have yet to be integrated (such as computational modeling approaches). Longitudinal brain research may also help towards better characterization of normative developmental trajectories, and the consequences for functional and structural brain development (see for instance an overview of normative structural development papers^52^). Eventually, these insights may help in understanding how for instance psychopathology may be explained as a deviation from normative development^53^. Given that longitudinal studies within the field of developmental cognitive neuroscience are time-consuming, valuable, and dependent on long-lasting research funding, the large longitudinal datasets that are becoming increasingly accessible (e.g.,^54–57^) will be important for advancing the field and examine the neurodevelopmental changes of learning.

In this review, we focused predominantly on individual learning. However, learning obviously does not happen in a vacuum, and humans learn the vast majority of their knowledge from other humans or are influenced by the *social context* of learning. Learning in a social context is hugely complex and encompasses interactions between learning and the regulatory demands of a social context (e.g., distraction by others), motivational processes (e.g., the desire to interact and engage with others), and our experience with others (e.g., learned trustworthiness of others). As such, an individual’s social context can impact the rate of learning, as well as what is learned in a given situation depending on the developmental period. For instance, reinforcement learning models have been used to study how we update our expectations about others across development and how this is distinct from non-social learning (e.g.,^58–63^). Although the social context of learning warrants a review article of itself, we do want to highlight that the methods displayed in the current review are also valuable to apply to research that studies learning in a social context.

Taken together, the study of behavioral learning can benefit from both structural and functional MRI research. We discussed methodological approaches that aim to unravel why learning interventions work, what learning computations change across development, and how learning difficulties are distinct between subgroups of individuals. These corresponding findings indicate that these approaches have the potential to have a lasting impact on promoting children’s and adolescents’ positive development.

### Reporting summary

Further information on research design is available in the Nature Research Reporting Summary linked to this article.

## Supplementary information

Reporting Summary Checklist

## Data Availability

No datasets were generated or analyzed during the current study.
